# Analytical Model for Blood Glucose Detection Using Electrical Impedance Spectroscopy

**DOI:** 10.3390/s20236928

**Published:** 2020-12-04

**Authors:** Bruna Gabriela Pedro, David William Cordeiro Marcôndes, Pedro Bertemes-Filho

**Affiliations:** Departamento de Engenharia Elétrica, Universidade do Estado de Santa Catarina, Joinville 89219-710, Santa Catarina, Brazil; bruna.pedro@edu.udesc.br (B.G.P.); david.wcm@edu.udesc.br (D.W.C.M.)

**Keywords:** blood glucose, analytical model, impedance spectroscopy, noninvasive monitoring

## Abstract

Pathogens and adulterants in human feeding consumables can be readily identified according to their electrical properties. Electrical bioimpedance analysis (BIA) has been widely used for body contents characterization, such as blood, urine, lactate, and sweat. If the concentration of glucose in blood alters the electrical properties of the blood medium, then the impedance spectrum obtained by BIA can be used to measure glycemia. For some applications, artificial neural networks allow the correlation of these parameters both impedance and concentration of glucose by means of symbolic and statistical rules. According to our literature review, there is not any physical model that allows the interpretation of the relationship between blood’s electrical properties from impedance spectra and the concentration of glucose in blood plasma. This article proposes a simplified physical model for blood electrical conductivity as a function of concentration of glucose, based on Bruggeman’s effective medium theory. The equations of this model were obtained considering an insulating phase distribution diffused in a conductive matrix, in which red blood cells are represented by macroscopic insulating nuclei and glucose molecules by microscopic insulating particles. The impedance spectrum for different glucose concentrations (4.0 to 6.8 mmol/L) in a blood sample, published by Kamat Bagul (2014), were compared to the proposed model. The results showed a significant correlation with the experimental data, showing a maximum error of 5.2%. The proposed model might be useful in the design of noninvasive blood glucose monitoring systems.

## 1. Introduction

Glucose is the main energy conveyor carbohydrate found in the most of animals. In humans, the concentration of glucose in blood may vary from 90 to 110 mg/dL [1] for healthy conditions. Any value that resides outside this range can be hazardous, causing a well known disease, called diabetes. Diabetes caused more than four million deaths in 2018 and is a leading global cause of heart disease, blindness, kidney failure, and lower-limb amputation [2]. For this reason, many monitoring techniques can be found in the literature which are specially designed to assist people with diabetes [3,4,5].

Most of the reliable diabetes monitoring systems are invasive that means they require a blood sample in order to measure the concentration of glucose. Unfortunately, these methods can cause pain, are expensive when used for continuous monitoring and prone to cause infections by opening wounds in the patient skin [6]. For that reason, developing non-invasive devices for accurate and reliable measure of concentration of glucose in blood is a remarkable topic in the specialized literature [7]. These devices can improve health quality of people with diabetes, allowing continuous monitoring of glucose levels and leading to a more precise administration of medication.

There are glucometers based on radio wave transmission. However, the higher frequencies necessary to reduce skin effects places difficulties in the measuring systems. Photoplethysmography is a method that relies upon infrared emission, where the absorption pattern is used to estimate glucose concentration. Unfortunately, this method requires a precise tracking of the heart rate by a second measuring system. In addition, an electrode pair must be placed on the skin in order to accurately measure tissue conductivity and permeability [8,9].

BIA has been successfully applied in the non invasive prognosis and characterization of living tissues. It consists of a pair of electrode placed on a patient’s skin. A sinusoidal current is injected by a pair of electrode, while the second pair measures the voltage drop across the sample under study. The tissue impedance can be calculated by using analytical models, and then biological parameters are estimated in order to assist medical prognosis [10]. The major drawback of BIA sensors are their complexity and development expensiveness [11]. In order to overcome that, ref. [8] proposes a reliable and low cost method whose results were compared with [11]. They got an equivalent precision into comparison to [11], presenting a good relationship between glucose concentrations and tissue impedance spectra.

Advances in electrical approaches are important for accurate detection, but analytical models have also been proven to be useful for blood investigations. There are models that analytically correlate blood proprieties with electrical parameters [1,6,12,13,14]. One such model establishes a correlation between blood and its mechanical proprieties, like viscosity, and electrical conductivity [12]. The results showed reasonable agreement with experimental data, for both diabetic and healthy blood, concluding that BIA can be used to investigate blood micro structure. The study presented by [13] brings a detailed model that includes several blood parameters and their relationship to electrical impedance. The study by [14] showed that red blood cell orientation and deformation can explain quantitatively the flow dependency of blood conductivity. Moreover, cell aggregation during blood sedimentation does also affect the electrical conductivity at different hematocrits [1]. Although these studies did not establish a clear connection between glucose concentration, excitation frequency and electrical blood conductivity, some experimental preliminary data have showed such a correlation of these parameters [8,11,15].

An alternative approach to the problem of estimating glucose levels is by using statistical and artificial neural networks (ANN). In these frameworks, an algorithm searches for a relationship between several input parameters and a target prediction: the blood glucose level. It is highly recommended that an ANN be trained with large experimental databases [16,17], so that it can lead to a proper convergence at its output. Once extensively trained and calibrated, ANN can then be used to estimate glucose levels with a given input data from outside the training set [18].

The main problem in any ANN comes from its output nature, that is, a statistical prediction [19]. ANN gives an output summed with an inference error that comes from its mathematical structure. The larger the training databases the better because, in order to reduce inference error, it is necessary to hold the input parameters in the training data neighborhood. On the contrary, a mathematical model that properly describes the phenomena gives a prediction that only relies upon measuring precision. Instead of the inference error, this approach carries a propagation error that arises only from the measuring methodology.

Regardless of its output prediction, ANN poses problems in the nature interpretation of the input data. In fact, the physical relationship between parameters are buried in the deep learning layers of ANN. If the relative rate of a parameter is required, then mathematical models are able to offer estimations by using straightforward algebraic equations. Additionally, ANN requires a new set of training data that depends upon a complete different experimental methodology in the search for such estimation. Therefore, ANN have less flexibility into comparison to mathematical models when a deep study of phenomena is required. In other words, to increase precision on the output of an ANN, it is necessary to enlarge the training databases, both quantitatively and qualitatively [20]. In contrast, mathematical models only require an increase in experimental precision in order to reduce the propagated error.

The study performed here applies the Effective Medium Theory (EMT) to describe the phenomena of glucose levels correlated to the electrical blood conductivity. The study done by [21] presents Bruggeman’s equations as an appropriate model for blood conduction, where the space’s nonhomogeneity distribution was taken into account. Thus, the volume conductor can be modeled by both insulating and conductive phases. The red cells and glucose molecules compose the insulating phase, while blood plasma the conductive one.

Firstly proposed by Bruggeman and Landauer [21], EMT has been the main framework in many applications where the macroscopically composed medium is the object under study [1,22,23,24,25,26]. EMT poses important technological applications like for percolation theory, employed for the monitoring and the simulation of oil reservoirs [27]. Another example deals with the analysis of carbon nanotubes conductivity, which is a material that increases performance of integrated circuits [28].

The focus of this article is to investigate the Bruggeman’s effective medium theory by developing and proposing a simplified physical model for blood electrical conductivity as a function of glucose concentration. It is expected that the proposed model may assist noninvasive glucose measuring devices and also be used as an alternative technique for ANNs. In addition, an analytical model can either be fully controlled regarding the error propagation or customized according to the parameters based on the device-individual setup.

## 2. Materials and Methods

**Figure 1:**
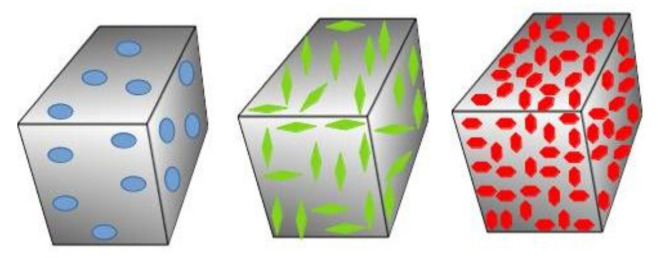
Representation of random insulating grain distributions in a conducting medium.

The EMT describes composite materials resistivity as a function of the relative phase concentration, shape, and distribution gradient. It is supposed that each ellipsoidal insulating grain is completely immersed in a conducting medium with known resistivity [26,29]. Figure 1 shows one randomly insulating grain distributions in a conducting medium [30].

The Bruggeman’s equations for asymmetric mediums, considering spherical grain inclusions, are:

(1) $$\frac{{\left(\sigma_{m}-\sigma_{l}\right)}^{3}}{\sigma_{m}}={\left(1-k\right)}^{3}\frac{{\left(\sigma_{h}-\sigma_{l}\right)}^{3}}{\sigma_{h}},$$

(2) $$\frac{{\left(\sigma_{m}-\sigma_{l}\right)}^{3}}{\sigma_{m}}={\left(1-\phi \right)}^{3}\frac{{\left(\sigma_{l}-\sigma_{h}\right)}^{3}}{\sigma_{l}}.$$

where $\sigma_{m}$ is the medium conductivity which contains two phases: a high conductivity material $\sigma_{h}$ and a low conductivity one ($\sigma_{l}$). The volumetric fraction of the high conductivity phase is represented by ϕ and *k* stands for the low one. This definition implies that ϕ=(1−k).

For this proposed model, $\sigma_{m}$ is considered the whole blood conductivity, $\sigma_{h}$ is the plasma conductivity, and $\sigma_{l}$ is the red cells conductivity. The volumetric fraction ϕ of the high conductivity phase is related to plasma volume, whereas *k* is related to red blood cells volume.

In ref. [21], Equation (1) represents a structure in which there is an insulating core submerged in a conductive medium with other smaller non-conductive particles. Consequently, Equation (1) is suitable for the required model, where the insulating core represents the red blood cells and glucose molecules the non conductive particles.

Considering a first approximation, $\sigma_{l}$ can be considered zero because red cells are insulating in the proposed model. This hypothesis applied in Equation (1) implies that:

(3) $$\sigma_{m}={\left(1-k\right)}^{\frac{3}{2}}\sigma_{h}.$$

It should be considered that blood vessels are represented by perfect cylinders with homogeneous conductivity. Therefore, at a constant temperature, the second Ohms’ law can be applied to give:

(4) $$R=\frac{L\rho }{A}$$

where ρ is the blood resistivity, *R* is the vessel resistance, and *L* and *A* are the length and traversal section area of the vessel, respectively. Combining Equations (3) and (4) and considering $\rho =\rho_{m}=\frac{1}{\sigma_{m}}$, Equation (4) can be redefined by:

(5) $$R=\frac{L}{A\sigma_{h}}\frac{1}{{\left(1-k\right)}^{\frac{3}{2}}}.$$

The impedance *Z* is the opposition to an alternate current flow. It is known that *Z* is highly sensitive to frequency for any living tissues. The real part of the impedance (*R*) is associated with water content, while the imaginary part (χ) with capacitive effects of cell membranes [31]. The modulus of the impedance can be given by:

(6) $$\left|Z\right|=\sqrt{R^{2}+\chi^{2}}.$$

While the imaginary part of *Z* (i.e., reactance) is given by:

(7) $$\chi =\frac{1}{2\pi fC}$$

where *C* is the total equivalent capacitance of the sample under study and *f* is the frequency excitation.

According to the classical electromagnetism theory, when charges are submitted to a potential variation, then a current flow without physical displacement of charge in a cross section between potential boundary is established. This phenomenon leads to a “resistance” associated with a capacitance, so that:

(8) $$C=\frac{\varepsilon \rho }{R},$$

where ε and ρ are the medium electrical permittivity and resistivity, respectively.

Combining Equations (5) and (8), and making $\rho =\rho_{m}=\frac{1}{\sigma_{m}}$, then Equation (8) can be rewritten as:

(9) $$C=\frac{A\varepsilon }{L}.$$

This last implies that the reactance given by Equation (7) can be written as:

(10) $$\chi =\frac{L}{2A\pi \varepsilon f}.$$

The electrical impedance can be rewritten combining Equations (9), (10) and (5) into (6), then the impedance modulus can be given by:

(11) $$\left|Z\right|=\frac{L}{A}\sqrt{\left(\frac{1}{\sigma_{h}^{2}}\frac{1}{{\left(1-k\right)}^{3}}\right)+\left(\frac{1}{4\pi^{2}\varepsilon^{2}}\frac{1}{f^{2}}\right)}.$$

The impedance modulus can also be expressed as function of the volumetric conductive fraction, so that:

(12) $$\left|Z\right|=\frac{L}{A}\sqrt{\left(\frac{1}{\sigma_{h}^{2}}\frac{1}{\phi^{3}}\right)+\left(\frac{1}{4\pi^{2}\varepsilon^{2}}\frac{1}{f^{2}}\right)}.$$

The analyzing expressions for volumetric conductive fraction take into account the composite material, the plasma, and the red cells, so that:

(13) $$V_{s}=V_{c}+Vi,$$

where $V_{s}$ is the blood volume (composite material), $V_{c}$ is the conductive material volume (plasma), and $V_{i}$ is the insulating material volume (red cells and glucose molecules). As already mentioned, the volumetric fraction of the high conductivity phase is given by:

(14) ϕ=1−k,

where $\phi =\frac{V_{c}}{V_{s}}$ and $k=\frac{V_{i}}{V_{s}}$.

These definitions are in full agreement with EMT, since the constants reinterpretation occurs less than a linear transformation over the same parameters. However, the volumetric fractions ϕ and *k* are not fixed, as their values change with glucose concentration. This hypothesis is in contradiction to EMT, once the blood vessel volume should also be considered fixed in this first model approach.

If one considers the cylindrical volume occupied by the plasma, glucose molecules, and red cells, then it can be assumed that red blood cells are much larger in volume and mass than glucose molecules and other ionic components present in blood. This implies that red cells have much larger inertia than plasma and glucose molecules. Therefore, it can be assumed that changes in glucose concentration only affect the volumetric fraction of plasma, which is represented here by ϕ. Then, it can be formulated from Equation (14) that:

(15) $$\phi -k^{\prime }=1-k$$

where $k^{\prime }$ is the volumetric fraction of glucose, *k* is the volumetric fraction only assigned to red blood cells, and ϕ is the volumetric fraction of plasma.

Combining Equations (12) and (15), the blood impedance modulus as a function of glucose concentration can be expressed by:

(16) $$\left|Z\right|=\frac{L}{A}\sqrt{\left(\frac{1}{\sigma_{h}^{2}}\frac{1}{{\left(1-k+k^{\prime }\right)}^{3}}\right)+\left(\frac{1}{4\pi^{2}\varepsilon^{2}}\frac{1}{f^{2}}\right)}$$

It is known that the electrical permittivity varies from one to another biological materials depending on the ions type and excitation frequency. This is also based on the polarization orientation caused by the application of a magnetic field to the material [32]. It is known that atoms, molecules, and defect of materials re-adjust to an equilibrium in response to an applied electric field. This re-adjustment of atoms, molecules, and defect of materials in response to an electric field is known as dielectric relaxation. The relaxation behavior depends on the lattice properties, frequency, and temperature [33]. Materials that exhibit a single relaxation time constant can be modeled by the Debye’s relation [32], which is given by:

(17) $$\varepsilon \left(\omega \right)=\varepsilon_{\infty }+\frac{\Delta \varepsilon }{\left(1-j\omega \tau \right)}$$

where $\omega =\frac{1}{2\pi f}$ is the angular frequency, $\varepsilon_{\infty }$ is the electrical permittivity at a high frequency, $\Delta \varepsilon (=\varepsilon_{s}-\varepsilon_{\infty }$), $\varepsilon_{s}$ is the static permittivity at a low frequency, and τ is the characteristic relaxation-time of the sample.

As a result, the proposed impedance modulus as a function of glucose concentration can be formulated by combining Equations (16) and (17), so that:

(18) $$\left|Z\right|=\sqrt{\left(\frac{L^{2}}{A^{2}}\frac{1}{\sigma_{h}^{2}}\frac{1}{\phi^{3}}\right)+\frac{{\left(L-j2\pi \tau Lf\right)}^{2}}{f^{2}{\left\lfloor 2\pi A\left(\varepsilon_{\infty }+\Delta \varepsilon \right)-4\pi^{2}A\varepsilon_{\infty }j\pi \tau f)+\right\rfloor }^{2}}}$$

## 3. Results

**Figure 2:**
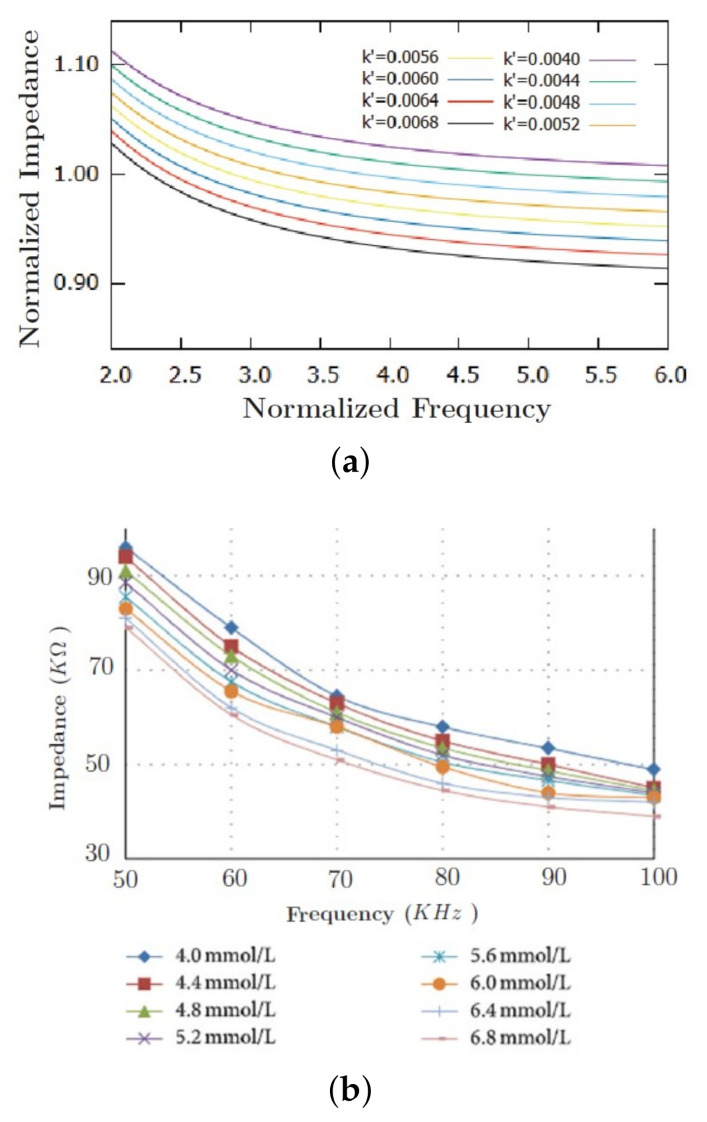
Normalized impedance spectra calculated by the proposed mathematical model (**a**) and the experimental data (**b**) presented by [8] (License CC BY 3.0 at https://creativecommons.org/licenses/by/3.0).

Figure 2a shows the impedance spectrum for different glucose concentrations. Equation (18) was normalized and rewritten in terms of $\sigma_{m}$, $\sigma_{h}$, L, A, $\varepsilon_{\infty }$, Δε, τ, and *f*. The new set of dimensionless constants are: $Z=Z/Z_{norm}$; $k=k/k_{norm}$; $k^{\prime }=k^{\prime }/k_{norm}^{\prime }$; $f=f/f_{norm}$. The constants *a*, *b*, *c*, *d* and *g* in Equation (19) represent a relative variation of *Z* in terms of blood volume, glucose concentration, and frequency, respectively. The factor $Z_{norm}$ is obtained by means of *L* and *A*, $k_{norm}^{\prime }$ by both glucose molar and volumetric densities and $f_{norm}$ by the blood’s electrical permittivity.

**Figure 3:**
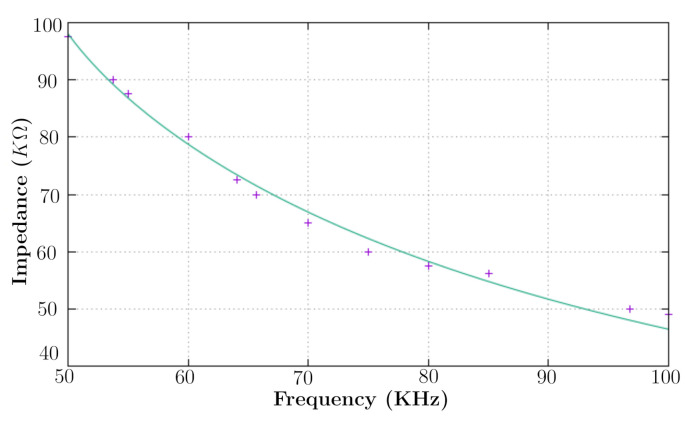
Frequency response of the modeled impedance data for a glucose concentration of 4.0 mmol/L from [8].

The impedance spectrum is calculated by varying the dimensionless glucose concentration factor $k^{\prime }$ from 0.0040 to 0.0068, as shown in Figure 2. The experimental data show a noticeable relation with the model proposed by Equation (18). Besides admensionality, Equation (18) can be fitted by experimental data. Equation (18) calculations were compared to the experimental data shown in [8] by using the least square method. According to Figure 3 at [8], the impedance was measured between both left hand and foot by using two AgCl electrodes connected the impedance converter AD5933. The glucose concentration used in this comparison was 4.0 mmol/L. As a result, Equation (18) was converted into a polynomial form as a function of frequency, such as:

(19) $$\left|Z\right|=\sqrt{b+\frac{{\left(a+cf\right)}^{2}}{f^{2}{\left(g+df\right)}^{2}}},$$

where a=L, $b=\frac{L}{A}\frac{1}{\sigma_{h}^{2}}\frac{1}{{\left(1-k+k^{\prime }\right)}^{3}}$, c=−2Lπjτ, $d=-4\pi^{2}A\varepsilon_{\infty }j\tau$ and $g=2\pi A(\varepsilon_{\infty }+\Delta \varepsilon )$.

Table 1 presents the errors associated in the fitting process, which are, in turn, the norm’s maximum errors at the least squares method.

Table 2 shows the deviations between modeled and experimental data, where the estimated constants in Table 1 were replaced in Equation (19). Notwithstanding, the model presents a major error at a frequency of 70 kHz. This corresponds to the apparent anomaly in Figure 2b, which might be due to a measuring error.

## 4. Discussion

It was shown in Figure 2 that the numerical curve has a significant similarity to the experimental one, implying that the proposed model can be used for modeling in vivo impedance data in a frequency range from 50 to 70 kHz. It was observed that the impedance module decrease exponentially with increasing frequency, whereas the error between data increases as increasing frequency. It can be noted in Figure 2b that the impedance modulus converges to approximately 45 kΩ (±5 kΩ) at 100 kHz, whereas, in Figure 2a, there is no data overlap even at high frequency. The discrepancy at high frequency observed in Figure 2b might be explained by the cell membrane capacitance which, in turn, is responsible for the transport of ions within the cell nucleus. Therefore, this imposes a limitation on the proposed numerical model at higher frequencies. Further investigations need to be done in order to find the optimal frequency range for this glucose measurement method.

It must be emphasized that the impedance variation due to glucose concentration is much smaller than the frequency excitation itself. Therefore, further investigations regarding the influences of other blood constituents (e.g., vitamins, lipids, lactate, amino acids, metabolic wastes and electrolytes) upon impedance spectra must be carefully done. However, regarding the low blood glucose sensitivity with respect to impedance, a similar result using occlusion spectroscopy was also found by [34]. In this study, the authors carried out in vitro experiments in a glucose range concentration from 0 and 100 mg/dL at different hematrocit concentrations. The glucose concentration used in the study [34] is much higher than the one we used for modeling, as it can be seen in Figure 2b. The study [34] verified that the glucose variation is quite insensitive to the occlusion spectroscopy intensity, whereas it is very significant to the percentages of hematrocit and the signal wavelength. As a result, the results found here and by [34] may denote a standard behavior of the human circulatory system, even using different measuring techniques.

Results at Figure 2b showed that the impedance spectra for glucose concentrations of 6.4 and 6.8 mmol/dL are quite different in comparison to other curves. This might suggest either a measuring procedure error or parasitic interference of the instrumentation used by [8] for collecting the data. This difference was confirmed by the proposed numerical model, then not allowing a proper prediction for this type of behavior.

A maximum error of 7.0% when calculating the coefficients from Equation (19) was found, which might be explained by the methodology adopted here for extracting the data from a plot-manual and visual inspection. We believe that having access to more experimental data in a wider glucose concentration range, both standard deviation and fitting errors, might be reduced. Regarding Table 2, the maximum impedance error of 5.2% at 100 kHz can also be explained by the non-idealities of the instrumentation used to measure the published data, especially at higher frequencies. Furthermore, this error could also be caused by the not standard visual-extraction technique used in this paper.

It is known that the resistance of any part of the tissue does not exceed 500 Ω in a short distance when measured by typical AgCl electrodes [6]. Although the results calculated here ranged from 40 to 100 kΩ, the impedance magnitudes seems to be plausible over the frequency range of 50 to 100 kHz for such an electrode arrangement used by [8]. It is known that body impedance depends on the body site, the size of electrode, and the measuring technique, which, in turn, used a bipolar technique (current is applied between the two electrodes and a voltage drop is measured across them). In a bipolar technique, the total measured impedance includes both body and electrode–skin interface impedance; then, it is expected to get higher impedance in comparison to the tetrapolar technique. Furthermore, the bigger the distance between electrodes, the higher its equivalent impedance will be, even using small-area electrodes. In addition, electrode–skin interface impedance plays a great role in the impedance sprectrum at lower frequencies, which might explain the fact that the work by [11] acknowledged that their results correlate better with capacitance change.

The fact that the impedance drops with increasing frequency certainly denotes a capacitive behavior, which is a typical characteristic of the dispersive medium like biological tissues. However, we proposed investigating the relative variation of impedance into respect to the concentration of glucose in blood. By deriving the Z final equation, one can observe that the impedance variation does not contain any capacitive element, especially in the frequency range used here. Although the behavior of the impedance curves seemed to correspond well to the data presented in both [8,11], care should be taken when using both different frequency range and electrode measuring technique.

It should bear in mind that our model is linked to the behavior of an impedance change related to a glycemic rate. Although the absolute value of the impedance has differences in comparison to other studies in the area, it is worth mentioning that, in both cases, the impedance change due to glucose is similar. The robustness of our proposal is mainly due to the linearized form utilized by the equations. As a future work, using different electrode configurations and types, we are preparing to collect a couple pieces of data from impedance spectra in blood in order to improve the accuracy of the mathematical modeling. We believe that local impedance spectra obtained from a 4-electrode system will differ from the one obtained by [8,11], and it also might be more sensitive to the interstitial glucose in the local blood stream.

**Figure 4:**
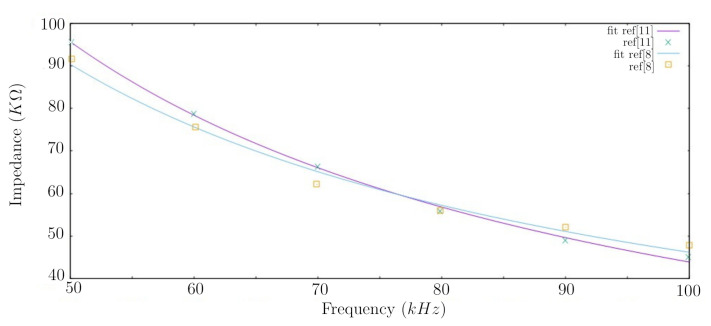
Modeled impedance spectra for a glucose concentration of 4.0 mmol/L from [8] and 4.1 mmol/L from [11].

Data from [11] were also used for further investigations in terms of robustness and efficiency. It can be observed that the proposed analytical model can also stand when data published by [11] is used, presenting smaller errors for the coefficients and predictions compared to the results presented in Table 2, as shown in Table 3. The smaller errors may suggest that the measuring technique and body site might affect the modeled impedance. In addition, the model seems to present a good sensitivity with respect to the impedance measurement technique, since the measurement location and the type of electrodes used in both experiments in [8,11] are different. Figure 4 shows the fitting data from modeling and their respective raw data from [8,11] for a similar concentration of glucose in blood.

Finally, the proposed analytical model may improve the performance and increase the accuracy of commercial glucometer devices. The equations presented here provided a crude understanding of the physics behind the blood conductivity, which sheds light on the dynamic behavior between the blood glucose concentration and its electrical parameters.

## 5. Conclusions

Regarding the mathematical relationship between blood glucose concentration and its electrical impedance, the BIA technique was presented as a suitable method for developing noninvasive glucose measuring systems into comparison to other noninvasive techniques. Moreover, the proposed mathematical modeling could confirm the validity of the hypothesis, where Bruggeman’s effective medium equation was satisfactorily used for modeling the blood conductivity as a function of its glucose concentration. Although the proposed model is simple, we could qualitatively explain the behavior of blood’s impedance with respect to its glucose concentration upon a short frequency range.

## Figures and Tables

**Table 1:** Coefficient values from Equation (19) and percentage errors with respect to data obtained from [8].

Coefficient	Value	Error %
a	$-1.12600\times 10^{0}$	4.0
b	$+5.07358\times 10^{-3}$	7.0
c	$+2.60469\times 10^{-2}$	4.0
d	$-5.64804\times 10^{-6}$	4.0
g	$+2.45902\times 10^{-4}$	4.0

**Table 2:** Comparison between calculated impedance from Equation (19) with the experimental one extracted from Figure 2b and their respective errors.

*f* (kHz)	$Z_{\mathrm{numerical}}$ (kΩ)	$Z_{\mathrm{experimental}}$ (kΩ)	Error %
50.0	97.9	97.5	0.5
55.0	86.8	87.5	0.8
60.0	78.7	80.0	1.6
65.6	71.6	70.0	2.2
70.0	66.9	65.0	2.9
75.0	62.3	60.0	3.8
80.0	58.3	57.5	1.4
85.0	54.8	56.2	2.6
96.0	48.0	50.0	4.0
100.0	46.4	49.0	5.2

**Table 3:** Coefficient values from Equation (19) and percentage errors with respect to data obtained from [11].

Coefficient	Value	Error %
a	−71,993.4 ×$10^{0}$	1.6
b	$+2.46206\times 10^{0}$	3.4
c	$+5.84262\times 10^{-2}$	1.6
d	$+5.87295\times 10^{-2}$	1.6
g	−72,208.6 ×$10^{0}$	1.6

## Abbreviations

The following abbreviations are used in this manuscript:

BIA	Bioimpedance Analysis
ANN	Artificial Neural Networks
EMT	Effective Medium Theory
